# Factors associated with in-hospital mortality in HIV-infected children treated for tuberculous meningitis hydrocephalus

**DOI:** 10.1007/s00381-023-06205-7

**Published:** 2023-11-15

**Authors:** Basil Enicker, Colleen Aldous

**Affiliations:** 1 Department of Neurosurgery, Inkosi Albert Luthuli Central Hospital, KwaZulu-Natal, Durban, South Africa; 2 Nelson R. Mandela School of Medicine, University of KwaZulu-Natal, 719 Umbilo Road, Congella, Durban, 4001 South Africa

**Keywords:** Endoscopic third ventriculostomy, Infarcts, Tuberculoma, Ventriculoperitoneal shunt

## Abstract

**Purpose:**

The study aimed to investigate factors associated with in-hospital mortality in children diagnosed with tuberculous meningitis (TBM) hydrocephalus and HIV co-infection undergoing cerebrospinal fluid diversion procedures and their complications.

**Methods:**

Data were collected retrospectively and prospectively between 2007 and 2022. Data collected included demographics, clinical characteristics, antiretroviral therapy (ART) status, biochemistry results, CD4 count, radiology findings, CSF diversion procedures (and complications), length of hospital stay (LOHS), and in-hospital mortality.

**Results:**

Thirty-one children were included, with a mean age of 6.7 ± 5.3 years and 67.7% males. Median admission Glasgow Coma Scale (GCS) was 11 (IQR 9–15). Hypertonia (64.5%) and seizures (51.6%) were frequently observed clinical characteristics. Sixty-one percent of children were on ART. Cerebral infarcts and extra-meningeal TB were diagnosed in 64.5% and 19.3% of cases, respectively. The median CD4 count was 151 (IQR 70–732) cells/µL. Surgical procedures included ventriculoperitoneal shunt (VPS) in 26 cases and endoscopic third ventriculostomy (ETV) in five children. VPS complication rate was 27%. No complications were reported for ETV. Median LOHS was 7 days (IQR 4–21). Eleven children (35.5%) died during admission. Factors associated with mortality included GCS (*p* = 0.032), infarcts (*p* = 0.004), extra-meningeal TB (*p* = 0.003), VPS infection (*p* = 0.018), low CD4 count (*p* = 0.009), and hyponatremia (*p* = 0.002). No statistically significant factors were associated with VPS complications.

**Conclusion:**

TBM hydrocephalus in HIV-infected children carries a high mortality. Clinicians in high-prevalence settings should have a high suspicion index and institute early treatment.

## Introduction

Tuberculous meningitis (TBM) is a frequently diagnosed central nervous system (CNS) infection in individuals living with HIV [1, 2]. HIV infection in children is particularly concerning, with high morbidity and mortality rates. In 2021 alone, it was estimated that approximately 110,000 children and adolescents died from AIDS-related pathologies, predominantly among those aged 10 years and younger [3]. The underlying immunosuppression in HIV-infected children predisposes them to increased mortality from CNS infections [2, 4].

The resurgence of TBM has been closely associated with the HIV/AIDS pandemic [4–6], with hydrocephalus detected in more than 80% of children with TBM [7–9]. The burden of disease related to TBM and HIV co-infection has been significant in South Africa [10]. However, the precise incidence of TBM-related hydrocephalus in HIV-infected children remains unknown. TBM leads to hydrocephalus through two main pathways. Firstly, cerebrospinal fluid (CSF) obstruction occurs due to accumulation of thick tuberculous exudate in the basal cisterns. Secondly, tuberculomas cause obstruction of the third ventricle, aqueduct, and fourth ventricle outlet foramina [5].

Management of TBM and HIV in children is challenging due to the necessity of treating both conditions simultaneously, which can result in adverse drug interactions and tuberculosis-associated immune reconstitution inflammatory syndrome (TB-IRIS), leading to increased morbidity and mortality [11]. In HIV infection, TBM can present with atypical CSF results, including normal CSF, further complicating diagnosis and potentially causing delays [12]. Delayed initiation of medical treatment can lead to long-term neuro-disabilities caused by various insults to the CNS, hydrocephalus, and increased intracranial pressure (ICP) [13]. Left untreated, hydrocephalus can lead to visual deterioration, coma, and even death.

Ventriculoperitoneal shunt (VPS) and endoscopic third ventriculostomy (ETV) are crucial surgical interventions for managing TBM hydrocephalus. However, the risk of complications associated with VPS is presumed high in HIV-infected patients due to their compromised immune status [14]. While previous studies have reported outcomes in the management of TBM hydrocephalus in HIV-infected patients, these studies have predominantly focused on adults [15–17]. Currently, no data exist regarding the outcomes of CSF diversion procedures, specifically VPS or ETV, in managing TBM-related hydrocephalus in HIV-infected children.

Therefore, this study aimed to investigate the factors associated with in-hospital mortality in HIV-infected children undergoing VPS and ETV procedures for treating TBM-related hydrocephalus. Additionally, we aimed to explore the factors related to complications arising from these CSF diversion procedures.

## Methods

The study used retrospective data from January 2007 to January 2017 and prospectively collected data from February 2018 to February 2022 at the Department of Neurosurgery (DoN), Inkosi Albert Luthuli Central Hospital, Durban, South Africa. The study population included HIV-infected children (birth to 17 years) diagnosed with TBM-related hydrocephalus who were referred for permanent CSF diversion. HIV-non-infected children or those with hydrocephalus caused by cryptococcal or acute bacterial meningitis were excluded.

Data collected encompassed various parameters, including age, sex, clinical characteristics, admission Glasgow Coma Scale (GCS), initiation of antiretroviral therapy (ART), admission laboratory investigations (full blood count, urea, and electrolytes), CD4 count, HIV viral load, CSF results, radiology findings, CSF diversion procedure, complications of the procedure, length of hospital stay (LOHS), in-hospital mortality, time to mortality, and Glasgow Outcome Scale (GOS) at discharge and 12 months follow-up. The GOS scores were classified as mild to moderate disability (GOS 4–5) and severe disability to mortality (GOS 1–3).

The HIV status of the children was confirmed using data from the national health laboratory services. TBM was diagnosed according to consensus criteria, categorized as definite, probable, or possible [18]. Following the diagnosis of TBM hydrocephalus, medical treatment was instituted by the pediatric infectious disease unit (PIDU), including therapeutic lumbar punctures (LPs), provided there were no clinical and radiological contraindications to LP. Indications for referral to the DoN for CSF diversion included severe hydrocephalus, obstructive hydrocephalus caused by tuberculomas, alteration in level of consciousness, deteriorating vision, and worsening neurological deficits.

A four drug anti-tuberculosis (TB) regimen was administered, which included isoniazid (20 mg/kg), rifampicin (20 mg/kg), pyrazinamide (40 mg/kg), and ethionamide (20 mg/kg) for 2 months (intensive phase), followed by 10 months of continuation phase treatment with isoniazid and rifampicin. The duration of treatment could be longer depending on response to therapy. Corticosteroids were used to minimize inflammation. Adjustment of ART to minimize potential drug interactions and toxicities was managed by the PIDU. Children not on ART were assessed for initiation, preferably within 4 to 8 weeks of starting anti-TB therapy [19, 20].

Detailed clinical examination and radiology imaging (chest X-ray, CT/MRI brain scan, and abdominal ultrasound when indicated) were conducted. The severity of TBM was assessed using the refined British Medical Research Council (BMRC) grading system [21]. The diagnosis of hydrocephalus was established based on CT or MRI brain scans, which revealed dilated ventricles, with or without loss of sulcal markings, temporal horns > 2 mm, trans-ependymal seepage, and associated clinical symptoms.

While differentiating between communicating and non-communicating (obstructive) hydrocephalus using CT/MRI brain scans alone is challenging, a proportionally dilated fourth ventricle on neuroimaging suggests the possibility of communicating hydrocephalus, unless an obvious tuberculoma is causing obstructive hydrocephalus. Although some centers use air encephalogram to distinguish between communicating and non-communicating hydrocephalus in the presence of a proportionally dilated fourth ventricle, this was not part of our protocol [22].

CSF diversion procedures, such as VPS insertion or ETV, were performed following standard guidelines [22, 23]. ETV was the preferred method in children with associated tuberculoma in the posterior fossa causing obstructive hydrocephalus. Following the procedure, all children were admitted to the high care ward for a minimum of 48 h of monitoring and transferred to general ward in stable condition. Follow-up assessments were conducted at the outpatient department after discharge.

Descriptive statistics were employed to summarize the demographic and clinical characteristics of the children. The numeric data’s normality was assessed; normally distributed variables were presented using the mean and standard deviation, while non-normally distributed variables were reported using the median and interquartile range (IQR). Chi-square tests were used to identify categorical factors associated with mortality, such as sex, clinical characteristics, initiation of ART, and radiological features (infarcts, tuberculoma, basal enhancement). *T*-tests or Mann–Whitney tests were utilized to compare numeric risk factors, including age, admission GCS, CD4 count, viral load, CSF, hematology, and biochemical results, between those who died and those who survived. Kaplan–Meier survival analysis was employed to evaluate time to death. The significance level was set at 0.05. The study was approved by the Biomedical Research Ethics Committee of the University of KwaZulu-Natal on the 8th of February 2018 (reference number BE607/17).

## Results

A total of 31 HIV-infected children were included in the study, with 11 (35.5%) children dying during the admission period. The demographic profiles are summarized in Table 1. The mean age was 6.7 ± 5.3 years, and majority of children were males (21; 67.7%). The median admission GCS was 11 (IQR 9–15). TBM diagnosis was definite in four (13%) children and probable in the remaining (27; 87%) (*p* = 0.601). The refined BMRC grades and clinical characteristics are presented in Table 1. The majority of children (19; 61%) were on ART.

**Table 1:** Demographic profiles and clinical characteristics of HIV-infected children diagnosed with TBM hydrocephalus, comparing children alive at discharge and those who died during hospitalization

**Variable**	**Total (*n* = 31)**	**Alive (*n* = 20; 64.5%)**	**Dead (*n* = 11; 35.5%)**	***p* value**
**Age, mean (SD)**	6.7 ± 5.3	7.2 ± 5.5	6 ± 5	0.521
• 0–6	19 (61.2%)	12 (60%)	7 (64%)	
• 7–12 • 13–17	6 (19.4%) 6 (19.4%)	3 (15%) 5 (25%)	3 (27%) 1 (9%)	0.568
**Sex**				
• **Male** • **Female**	21 (67.7%) 10 (32.3%)	12 (60%) 8 (40%)	9 (81.8%) 2 (18.2%)	0.262
**Admission GCS (median, IQR)**	11 (9–15)	11.5 (10–15)	9 (6.5–11.5)	0.032
**Refined BMRC grade**				
• Grade 1	2 (6.5%)	2 (10%)	0 (0%)	
• Grade 2a	8 (26%)	7 (35%)	1 (9%)	0.113
• Grade 2b	7 (22.5%)	5 (25%)	2 (18%)	
• Grade 3	14 (45%)	6 (30%)	8 (73%)	
**Clinical characteristics**				
• Hypertonia	20 (64.5%)	12 (60%)	8 (72.7%)	0.698
• Seizures	16 (51.6%)	9 (45%)	7 (63.6%)	0.458
• Headaches	15 (48.4%)	9 (45%)	6 (54.5%)	0.716
• Cranial nerve deficits	7 (22.6%)	5 (25%)	2 (18.2%)	1.000
• Hemiparesis	8 (25.8%)	5 (25%)	3 (27.3%)	1.000
• Vomiting	7 (22.6%)	3 (15%)	4 (36.4%)	0.210
• Poor appetite	5 (16.1%)	4 (20%)	1 (9.1%)	0.631
• Meningism	10 (32.3%)	4 (20%)	6 (54.5%)	0.106
**ART**				
• Yes • No	19 (61%) 12 (39%)	13 (65%) 7(35%)	6 (54.5%) 5 (45.5%)	0.705

*ART* anti-retroviral therapy, *BMRC* British Medical Research Council, *GCS* Glasgow Coma Scale, *IQR* interquartile range, *SD* standard deviation, *TB* tuberculosis

**Fig. 1:**
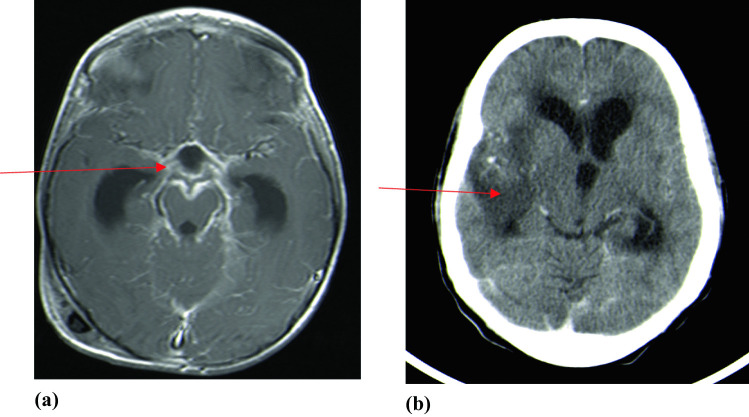
**a** T1-weighted post contrast MRI brain scan showing basal enhancement and **b** post contrast CT brain scan showing right middle cerebral artery territory infarct

**Fig. 2:**
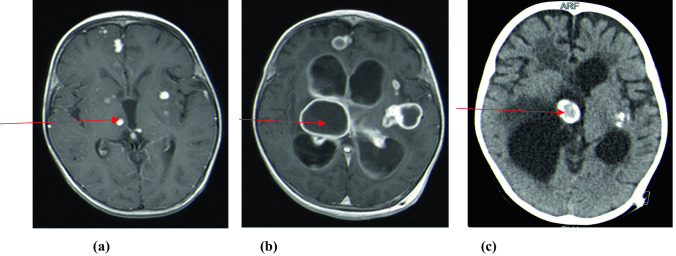
**a** T1-weighted post contrast MRI brain scan showing multiple tuberculomas in a 4-year-old child on anti-TB therapy and ART. The tuberculomas increased in size (paradoxical TB-IRIS) while the child was on anti-TB therapy and ART (**b**). A non-contrast CT brain (**c**) performed at 12 months follow-up showed calcification of tuberculomas

**Fig. 3:**
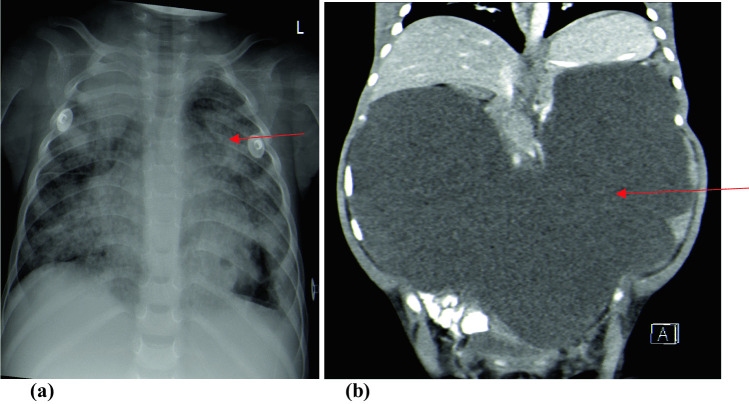
Chest X-ray of child with TB pneumonia (**a**) and abdominal CT showing ascites in a child diagnosed with TB abdomen (**b**)

The radiological findings, CSF diversion procedures, and their complications are shown in Table 2. Basal enhancement (90.3%) and infarcts (64.5%) were the most common neuroradiological findings (Fig. 1a and b, respectively). Fourteen (45.2%) children were diagnosed with tuberculomas on CT/MRI brain scans, with one child experiencing an increase in tuberculoma size (paradoxical TB-IRIS) while on anti-TB therapy, steroids and ART (Fig. 2a–c). The child had a prolonged admission period of 92 days, responded to treatment, however, had residual severe neurocognitive deficits. Among the 14 children with tuberculomas, nine (64%) were on ART, while five (36%) were not (*p* = 0.756). Extra-meningeal TB was diagnosed in six (19.3%) children, and TB pneumonia was diagnosed in four children (Fig. 3a). Two children developed TB abdomen, complicated by ascites (Fig. 3b). All children with extra-meningeal disease died (*p* = 0.003).

**Table 2:** Radiological findings and surgical management of HIV-infected children diagnosed with TBM hydrocephalus, comparing children alive at discharge and those who died during hospitalization

**Variable**	**Total (*n* = 31)**	**Alive (*n* = 20; 64.5%)**	**Dead (*n* = 11; 35.5%)**	***p* value**
**CT brain findings**				
• Basal enhancement	28 (90.3%)	17 (85%)	11 (100%)	0.290
• Infarcts	20 (64.5%)	9 (45%)	11 (100%)	0.004
• Tuberculoma	14 (45.2%)	10 (50%)	4 (36.4%)	0.707
**Extra meningeal TB** • TB pneumonia • TB abdomen	6 (19.3%) 4 (13%) 2 (6.5%)	0 0 0	6 (54.5%) 4 (36.4%) 2 (18.2%)	0.003 0.010 0.118
**VPS procedure**	26 (84%)	16 (80%)	10 (91%)	0.631
• **Shunt type**				
o **AIS** o **Non-AIS**	21 (81%) 5 (19%)	15 (94%) 1 (6%)	6 (60%) 4 (40%)	0.055
**VPS complication (in those who had shunt *n* = 26)** • Time to shunt complication, days (median, IQR)	7 (27%) 48 (25–84)	2 (12.5%) 205 (54–357)	5 (50%) 43 (25–48)	0.069 0.190
**VPS infection (in those who had shunt *n* = 26)**	6 (23%)	1 (6.3%)	5 (50%)	0.018
**ETV**	5 (16%)	4 (20%)	1 (9%)	0.631
**Length of hospital stay (days), median, IQR**	7 (4–21)	6 (4–8)	22 (4–50)	0.095

*AIS* antibiotic impregnated shunt, *CT* computerized tomography, *ETV* endoscopic third ventriculostomy, *IQR* interquartile range, *TB* tuberculosis, *VPS* ventriculoperitoneal shunt

The median CD4 count was 151 (IQR 70–732) cells/µL, and the median viral load was 7449 (370–81,441) copies/mL. The median hemoglobin was 10.3 g/dL (9.1–12.2), and mean serum sodium was 133 ± 7. The mortality group had a mean sodium level of 128 ± 6.7 mmol/L, significantly lower than the group that survived (*p* = 0.002). The remaining laboratory findings are presented in Table 3.

**Table 3:** Association between laboratory results at admission for surgery and mortality

**Variable**	**Total (*n* = 31)**	**Alive (*n* = 20, 64.5%)**	**Dead (*n* = 11, 35.5%)**	***p* value**
Admission CD4 count cell/mL (median, IQR)	151 (70–732)	535 (124–756)	100 (22–202)	0.009
Viral load, copies/mL, (median, IQR)	7449 (370–81441)	3349 (236–77,721)	11,751 (890–81823)	0.310
Hemoglobin (g/dl)	10.3 (9.1–12.2)	10.3 (8.8–12.4)	10.2 (9.1–12)	1.000
White cell count (× 10^9^/L), IQR	7.9 (6.2–10.2)	7.7 (5.9–10.1)	9.0 (6.2–10.2)	0.403
Platelets, count (× 10^9^/L), IQR	422 (332–539)	423 (323–515)	413 (326–601)	0.555
Polymorphs (cells X 10^6^/L), IQR	24 (8–44)	26 (9–46)	23 (4–38)	0.919
Lymphocytes (cells X 10^6^/L), median, IQR	48 (22–70)	54 (34–72)	32 (18–70)	0.289
Protein g/L, median IQR	1.33 (1.07–2.08)	1.2 (0.8–2.1)	1.7 (1.2–2.5)	0.133
Chloride (mmol/L), IQR	112 (106–120)	113 (110–124)	110 (100–118)	0.197
CSF glucose (mmol/L), median, IQR	1.7 (1.3–2.0)	1.4 (1.2–2.0)	1.8 (1.4–2.0)	0.317
Sodium (mmol/L) mean ± SD	133 ± 7	136 ± 5	128 ± 7	0.002
Potassium (mmol/L), mean ± SD	4.2 ± 0.7	4.3 ± 0.7	4.1 ± 0.6	0.629
Urea (mmol/L), mean ± SD	4.5 ± 4.7	5.3 ± 5.6	3.0 ± 1.6	0.207
Creatinine (mmol/L), mean ± SD	32 ± 22	36 ± 25	26 ± 13	0.240

*IQR* interquartile range, *SD* standard deviation

**Fig. 4:**
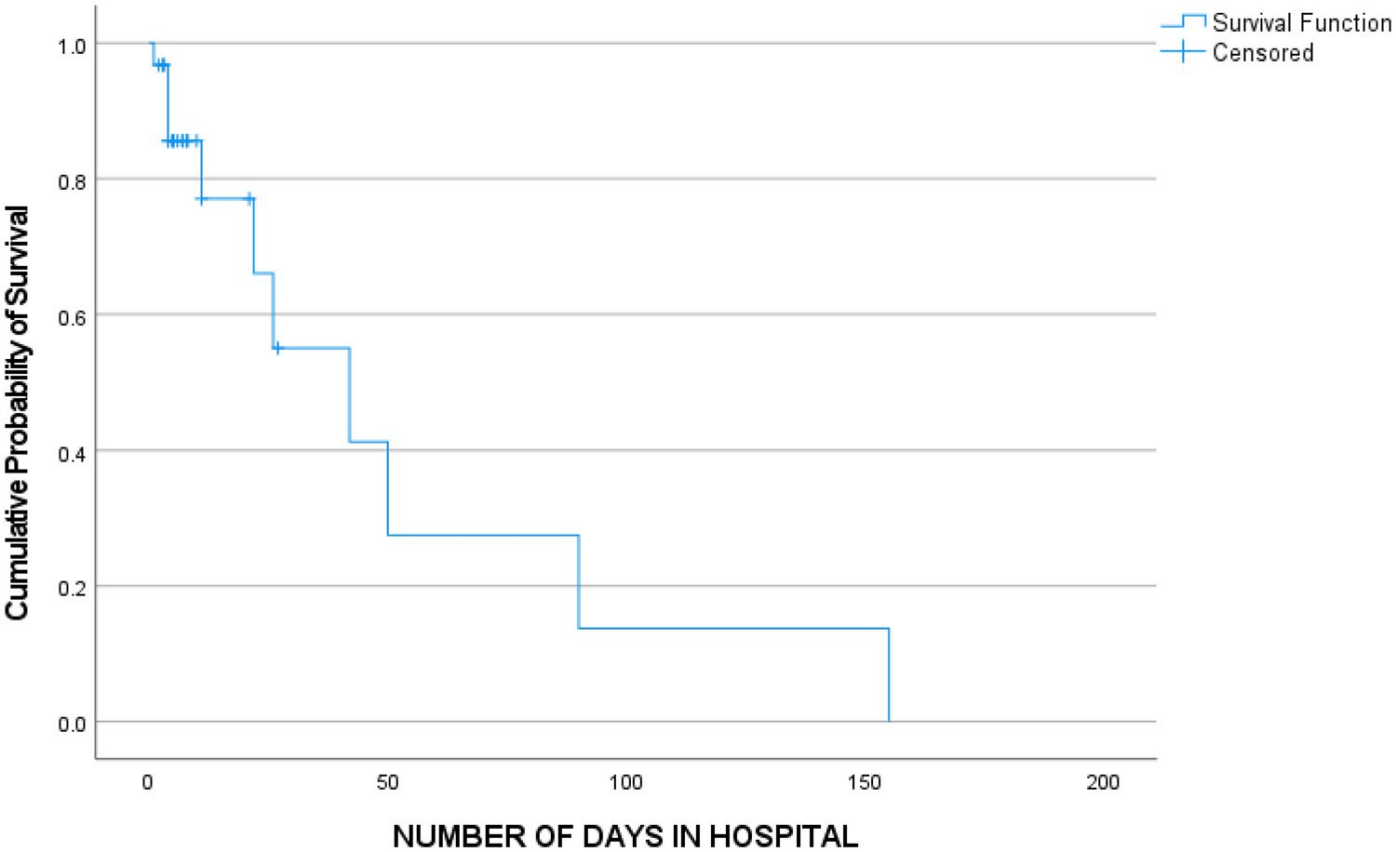
Kaplan–Meier plot of time to in-hospital mortality (*n* = 31). The overall median time to in-hospital mortality was 42 days

VPS procedures were performed in 26 (84%) children, while five (16%) underwent ETV. Seven (27%) VPS complicated, while there were no complications related to ETV. Six VPS complications were associated with infection, with five infections occurring in the mortality group (*p* = 0.018). Among the VPS infections in the mortality group, three were culture negative, while the other two cultured *Staphylococcus aureus* and *Proteus mirabilis* (both in the same child) and *Viridans streptococcus*. The analysis of factors associated with VPS complications is shown in Tables 4 and 5. Figure 4 presents the overall time to in-hospital mortality using a Kaplan–Meier plot.

**Table 4:** Analysis of key factors and their association with VPS complications

**Variable**	**Total (*n* = 26)**	**No VPS complication (*n* = 19; 73%)**	**VPS complication (*n* = 7; 27%)**	***p* value**
**Age, mean (SD)**	5.9 (5.1)	6.7 (5.5)	3.7 (2.9)	0.196
• 0–6	19 (73.1%)	13 (68.4%)	6 (85.7%)	
• 7–12 • 13–17	3 (11.5%) 4 (15.4%)	2 (10.5%) 4 (21.1%)	1 (14.3%) 0 (0%)	0.517
**Sex**				
• **Male** • **Female**	17 (65.4%) 9 (34.6%)	12 (63.2%) 7 (36.8%)	5 (71.4%) 2 (28.6%)	1.000
**Admission GCS (median, IQR)**	11 (8–13)	10 (7–13)	11 (8–15)	0.572
**Refined BMRC grade**				
• Grade 2a	6 (23.1%)	4 (21.1%)	2 (28.6%)	1.000
• Grade 2b	7 (26.9%)	5 (26.3%)	2 (28.6%)	
• Grade 3	13 (50%)	10 (52.6%)	3 (42.9%)	
**TB diagnosis**				
• Definite	2 (7.7%)	1 (5.3%)	1 (14.3%)	
• Probable	24 (92.3%)	18 (94.7%)	6 (85.7%)	1.000
**Radiological findings**				
• Basal enhancement	26 (100%)	19 (100%)	7 (100%)	-
• Infarcts	19 (73.1%)	12 (63.2%)	7 (100%)	0.134
• Tuberculoma	9 (34.6%)	5 (26.3%)	4 (57.1%)	0.188
**Extra-meningeal TB** • TB pneumonia • TB abdomen	5 (19.2%) 4(15.4%) 2 (7.7%)	2 (10.5%) 2 (10.5%) 0 (0%)	3 (42.9%) 2 (28.6%) 2 (28.6%)	0.101 0.546 0.065
**VPS type**				
• **AIS** • **Non-AIS**	5 (19.2%) 21 (80.8%)	2 (10.5%) 17 (89.5%)	3 (42.9%) 4 (57.1%)	0.101
**ART**				
• Yes • No	17 (65.4%) 9 (34.6%)	11 (57.9%) 8 (42.1%)	6 (85.7%) 1 (14.3%)	0.357

*AIS* antibiotic impregnated shunt, *CT* computerized tomography, *ETV* endoscopic third ventriculostomy, *IQR* interquartile range, *TB* tuberculosis, *VPS* ventriculoperitoneal shunt

**Table 5:** Association between laboratory results at admission for surgery and shunt complications

**Variable**	**Total (*n* = 26)**	**No VPS complication (*n* = 19; 73%)**	**VPS complication (*n* = 7; 27%)**	***p* value**
Admission CD4 count cell/mL (median, IQR)	177 (100–701)	202 (70–732)	151 (100–399)	0.611
Viral load, copies/mL, (median, IQR)	12,050 (370–81,823)	11,401 (241–74,000)	81,441 (890–574,712)	0.185
Hemoglobin (g/dl)	10.3 (8.6–12)	10.3 (8.5–12.5)	10 (8.6–11.8)	0.534
White cell count (× 10^9^/L), IQR	7.6 (6–10.2)	7.89 (6.2–10.2)	6 (4.0–10.76)	0.279
Platelets, count (× 10^9^/L), IQR	418 (332–521)	413 (318–460)	539 (363–601)	0.120
Polymorphs (cells X 10^6^/L), IQR	28 (10–48)	31 (16–48)	22 (4–64)	0.497
Lymphocytes (cells X 10^6^/L), median, IQR	54 (22–77)	62 (32–88)	36 (22–52)	0.169
Protein g/L, median IQR	1.41 (1.1–2.09)	1.4 (1.07–2.08)	1.54 (1.1–3.14)	0.534
Chloride (mmol/L), IQR	110 (106–118)	110 (106–117)	113 (100–128)	0.306
CSF Glucose (mmol/L), median, IQR	1.8 (1.3–2.0)	1.5 (1.3–2.0)	2.0 (1.7–2.1)	0.120
Sodium (mmol/L) mean ± SD	132 (7)	133 (7)	131 (7)	0.681
Potassium (mmol/L), mean ± SD	4.2 (0.7)	4.2 (0.7)	4.2 (0.6)	0.815
Urea (mmol/L), mean ± SD	4.5 (5.1)	5.1 (5.8)	2.9 (1.2)	0.336
Creatinine (mmol/L), mean ± SD	31 (22)	32 (25)	26 (6)	0.503

*IQR* interquartile range, *SD* standard deviation

Regarding children alive at discharge, six (30%) had mild to moderate disability, while 14 (70%) had severe disability. The median follow-up period was 15.5 months (IQR 3–91.3). Twelve (60%) of the 20 children alive at discharge were followed up for at least 12 months, with four (33%) experiencing mild to moderate disability and eight (67%) having severe disability. The median CD4 count at the last follow-up was 736.5 cells/µL (IQR 615–1265.3) for the eight children followed up for 12 months or more.

Univariate analysis of baseline characteristics (Tables 1 and 2) showed lower admission GCS, infarcts, extra-meningeal TB, VPS infection, lower CD4 count, and hyponatremia as factors trending towards association with mortality. Univariate analysis of baseline characteristics did not identify statistically significant factors associated with VP shunt complications (Tables 4 and 5), although there was a trend observed in children diagnosed with TB abdomen.

## Discussion

This is the first pediatric series giving the results of HIV-infected children treated for TBM hydrocephalus in the literature, such as rates of in-hospital mortality and VPS complications.

Our study reported an in-hospital mortality rate of 35.5%, which is lower than the reported rates in cohorts of shunted adult series [15, 16]. Sharma et al. reported a mortality rate of 66.7%, with 57% of deaths occurring within the first month; however, ART status was not documented [16]. Nadvi et al. reported a similar mortality rate of 66.7%, and none of the patients was on ART [15]. Access to ART, including improved TB and HIV treatment strategies over the years in South Africa, would explain the lower mortality in our series when compared to Nadvi et al.

Harrichandparsad et al. reported a mortality rate of 26.7% in patients with TBM-related hydrocephalus on ART, compared to historical controls who were not on ART, with a mortality rate of 66.7% [17]. In our study, only 61% of children were on ART, and 45.5% of the mortalities were not on ART; however, this difference was not statistically significant. In South Africa, ART was only initiated in individuals with a CD4 count below 200 cells/µL, but since 2016, all HIV-infected individuals are eligible for ART regardless of CD4 count [24, 25]. In our study, low CD4 counts were associated with in-hospital mortality but not with VPS complications, possibly due to the small sample size of VPS complications. Other authors reported no correlation between CD4 count and poor outcomes in shunted HIV-infected adult patients [16, 17].

Seizures were diagnosed in 51.6% of children, occurring in 63.6% of those who died; however, this was not statistically significant. Seizures increase cerebral metabolic rate, inducing oxygen insufficiency which prevents aerobic glycolysis and oxidative phosphorylation. The end results are worsening ischemia and cellular death [22].

In our study, hyponatremia was found to be associated with in-hospital mortality. Hyponatremia can be due to syndrome of inappropriate antidiuretic hormone (SIADH) secretion or cerebral salt-wasting syndrome [26]. Hyponatremia exacerbates cerebral edema, increasing susceptibility to seizures and poor outcomes. It is crucial to differentiate between SIADH and cerebral salt-wasting syndrome in order to ensure appropriate fluid and intravascular volume management.

Hemoglobin (Hb) levels were not found to be predictors of mortality in our study. Karande et al. reported lower Hb levels (less than 8 g/dl) in eight HIV-infected children, with a mortality rate of 12.5% [27], while another study found no differences in Hb levels between HIV-infected and non-infected children [28]. Hb plays a pivotal role in oxygen delivery to the brain, especially in the context of TBM hydrocephalus, due to susceptibility of the brain to ischemia from increased ICP and vasculitis.

Marais et al. reported BMRC grades 2 and 3 to be significant predictors of mortality in both HIV-infected and non-infected patients; however, our study did not confirm statistical significance in this regard [29]. Definite TB was diagnosed in 13% of children in our study. The culture of *Mycobacterium tuberculosis* (Mtb) from the CSF is considered the gold standard; however, the rate of positive culture varies in the literature. For instance, Bang et al. reported definite TB in 6% of children, while the remaining cases were categorized as probable (66%) and possible (28%) [30]. Grobbelaar et al. reported Mtb culture positivity rates of 17% and 56.4%, respectively [31, 32]. A Durban study on HIV-infected adults with TBM reported definite TB in 15.5% of patients [33].

Enhancing the diagnostic yield requires increased CSF submission volumes, laboratory infrastructure, expertise, and molecular tests such as GeneXpert MTB/RIF®, offering rapid results, improved sensitivity and specificity. The low rate of microbiological confirmation of Mtb in our study reflects the challenges confronting researchers in LMICs with the highest burden of HIV and TBM.

Cerebral infarcts, reported in 64.5% of children in our study, were associated with mortality. Infarcts are caused by vasculitis and intimal proliferation of cerebral arteries, commonly affecting areas such as the caudate nucleus, internal capsule, and thalamus [22]. This explains the documented hypertonia and hemiparesis in our series. Rohwlink et al. reported a 66% rate of infarcts and a mortality rate of 16% in a series of 44 children with tuberculous hydrocephalus [34]. The delayed resolution of cerebral exudate leads to ongoing inflammatory responses, even after initiating anti-TB medication, resulting in delayed infarcts [35]. Clemente Morgado et al., in a study of adult patients with TBM hydrocephalus, reported infarcts in 45.5% of patients, with 100% mortality in the HIV-infected group [36]. TB exudates can also infiltrate cranial nerves, resulting in deficits, as reported in 22.6% of children in our study [37].

Intracranial tuberculomas are frequently diagnosed pathology in HIV-infected children. Nevertheless, tuberculomas were not associated with mortality in our study. Notably, one child was diagnosed with neurological paradoxical TB-IRIS, characterized by progressive enlargement of tuberculomas, while on anti-TB therapy and ART. This occurred despite the child being on steroids, a phenomenon reported by other authors as well [11]. TB-IRIS manifests when initiation of ARTs occurs within a period ranging from 2 weeks to 3 months after commencement of anti-TB therapy [38]. It is postulated that ARTs result in restoration of the immune response to Mtb. Consequently, this immune resurgence, marked by an increase in CD4 T cell count, triggers excessive production of pro-inflammatory cytokines. Paradoxical TB-IRIS is differentiated from unmasking TB-IRIS, which refers to TB that only becomes clinically evident after initiation of ART [38, 39].

Risk factors for TB-IRIS include low CD4 count, high viral load, disseminated TB, increased CSF neutrophil count, and positive Mtb culture in the CSF [39]. The spectrum of neurological TB-IRIS can include worsening TBM, intracranial TB abscess, spinal epidural abscess, and TB radiculomyelitis [38, 39].

Corticosteroids are recommended therapy. It is advisable to continue ART during TB-IRIS episodes, as discontinuation may result in drug resistance. However, instances where there is depressed level of consciousness or severe disease nonresponsive to corticosteroids, discontinuation of ART should be considered [38, 39]. Marias et al. reported a 13% mortality in a series of 16 HIV-infected adults with TB-IRIS, while van Toorn et al. reported a single fatality within a series of four HIV-infected children diagnosed with TB-IRIS [40].

Indications for resection of tuberculomas include progressive enlargement, inducing mass effect and visual deterioration despite adequate medical treatment, particularly in posterior fossa [41]

Proactive management and prevention of HIV in children assume a critical role in reducing CNS infections and their complications. Successful prevention of mother-to-child transmission (PMTCT) programs in South Africa has precipitated a decline in the number of children born with HIV [42].

Effective management of communicating TBM hydrocephalus primarily involves anti-TB therapy, acetazolamide, corticosteroids, and frequent LPs. However, successful outcomes require strict adherence to treatment protocols, close monitoring, and follow-up [22]. Debates still persist regarding the optimal duration of anti-TB therapy for TBM-diagnosed patients [43].

Surgical treatment options vary from temporary measures such as external ventricular drain (EVD) placement to alleviate ICP, to permanent procedures, namely VPS or ETV [22]. Agrawal et al. recommended VPS for children with Palur grade II to III and EVD for grade IV, with progression to VPS if there is clinical improvement [44]. None of the children in our study underwent a trial of EVD placement. All children underwent early permanent CSF diversion, thus allowing focus on the medical management of TBM and its complications. Majority of children (84%) in our study were treated with VPS, and the shunt complication rate was 27%, which falls within the reported range of 10 to 40% [13, 44–47].

Mortality rates are notably elevated in HIV-infected individuals diagnosed with TB meningitis and concurrent secondary bacterial meningitis [2]. This observation elucidates the association between mortality and VPS infections in the current study.

ETV was performed in five children who had associated tuberculomas in the posterior fossa causing obstructive hydrocephalus. Some experts hold the view that ETV should be offered even in patients with communicating hydrocephalus; however, our approach remains selective [22]. ETV can convert non-communicating to communicating hydrocephalus thereby circumventing VPS-related complications. Figaji et al. reported an ETV success rate of 41% [48], while other studies reported success rates ranging from 59 to 65.4% [13, 49]. Performing ETV in the acute phase can be challenging due to the distortion of the anatomy of the third ventricle, thickened floor, and basal exudates. Some authors have suggested performing ETV during the chronic phase of TBM to enhance the prospects of success [50]. At the 12-month follow-up, 67% of children in our study were assessed as having a poor outcome, while adult series reported poor outcomes in 64.7% and 76.2% of cases, respectively [15, 16].

## Limitations of the study

Our study has certain limitations that should be acknowledged. First, the sample size was relatively small, which may have limited the statistical power to detect significant associations and generalize the findings to larger populations. Additionally, the lack of a comparison group of HIV-non-infected children prevented us from directly comparing outcomes between the two groups. Future studies with larger sample sizes and the inclusion of a control group are warranted to elucidate further the impact of HIV infection on the outcomes of children with TBM hydrocephalus.

The data were obtained from a single institution, which may introduce institutional biases and limit the generalizability of the findings to other settings. Multi-center studies involving different geographic locations and diverse populations would provide a more comprehensive understanding of the outcomes in this patient population. Despite these limitations, our study provides important insights into the outcomes of HIV-infected children undergoing treatment for TBM-related hydrocephalus.

## Conclusion

Our study highlights the high mortality associated with TBM-related hydrocephalus in HIV-infected children and complexities of managing this high-risk population. While the risk factors for mortality were elucidated, no statistically significant factors were found to be associated with VPS complications. Our findings underscore the urgent need for improved strategies for managing TBM-related hydrocephalus in HIV-infected children.

## Data Availability

The datasets collected and analyzed during the current study are available from the corresponding author on request.

## References

[1] Marais S, Pepper DJ, Schutz C, Wilkinson RJ, Meintjes G (2011). Presentation and outcome of tuberculous meningitis in a high HIV prevalence setting. PLoS ONE.

[2] Tenforde MW, Gertz AM, Lawrence DS, Wills NK, Guthrie BL, Farquhar C, Jarvis JN (2020). Mortality from HIV-associated meningitis in sub-Saharan Africa: a systematic review and meta-analysis. J Int AIDS Soc.

[3] Global and regional trends. https://data.unicef.org/topic/hivaids/global-regional-trends/. (Last accessed 4 May 2023)

[4] Fry SH, Barnabas SL, Cotton MF (2019). Tuberculosis and HIV-an update on the “cursed duet” in children. Front Pediatr.

[5] Schaaf HS, Seddon JA (2021). Management of tuberculous meningitis in children. Paediatr Int Child Health.

[6] Glynn JR (1998). Resurgence of tuberculosis and the impact of HIV infection. Br Med Bull.

[7] Chiang SS, Khan FA, Milstein MB, Tolman AW, Benedetti A, Starke JR (2014). Treatment outcomes of childhood tuberculous meningitis: a systematic review and meta-analysis. Lancet Infect Dis.

[8] Paliwal VK, Garg RK (2021). Hydrocephalus in tuberculous meningitis - pearls and nuances. Neurol India.

[9] Aulakh R, Chopra S (2018). Pediatric tubercular meningitis: a review. J Pediatr Neurosci.

[10] Walters E, Cotton MF, Rabie H, Schaaf HS, Walters LO, Marais BJ (2008). Clinical presentation and outcome of tuberculosis in human immunodeficiency virus infected children on antiretroviral therapy. BMC Pediatr.

[11] Marais S, Meintjes G, Pepper DJ, Dodd LE, Schutz C, Ismail Z (2013). Frequency, severity, and prediction of tuberculous meningitis immune reconstitution inflammatory syndrome. Clin Infect Dis.

[12] Pormohammad A, Nasiri MJ, Riahi SM, Fallah F (2018). Human immunodeficiency virus in patients with tuberculous meningitis: systematic review and meta-analysis. Trop Med Int Health.

[13] Chalasani R, Goonathilake MR, Waqar S, George S, Jean-Baptiste W, Yusuf Ali A (2022). The outcome of surgical intervention (ventriculoperitoneal shunt and endoscopic third ventriculostomy) in patients with hydrocephalus secondary to tuberculous meningitis: a systematic review. Cureus.

[14] Loan JJM, Poon MTC, Tominey S, Mankahla N, Meintjes G, Fieggen AG (2020). Ventriculoperitoneal shunt insertion in human immunodeficiency virus infected adults: a systematic review and meta-analysis. BMC Neurol.

[15] Nadvi SS, Nathoo N, Annamalai K, van Dellen JR, Bhigjee AI (2000) Role of cerebrospinal fluid shunting for human immunodeficiency virus-positive patients with tuberculous meningitis and hydrocephalus. Neurosurgery 47:644–649. discussion 649–650. 10.1097/00006123-200009000-00024 10.1097/00006123-200009000-00024 10981752

[16] Sharma RM, Pruthi N, Arimappamagan A, Somanna S, Devi BI, Pandey P (2015). Tubercular meningitis with hydrocephalus with HIV co-infection: role of cerebrospinal fluid diversion procedures. J Neurosurg.

[17] Harrichandparsad R, Nadvi SS, Suleman Moosa MY, Rikus van Dellen J (2019) Outcome of ventriculoperitoneal shunt surgery in human immunodeficiency virus-positive patients on combination antiretroviral therapy with tuberculosis meningitis and hydrocephalus. World Neurosurg 123:e574–e580. 10.1016/j.wneu.2018.11.221 10.1016/j.wneu.2018.11.221 30529520

[18] Marais S, Thwaites G, Schoeman JF, Török ME, Misra UK, Prasad K (2010). Tuberculous meningitis: a uniform case definition for use in clinical research. Lancet Infect Dis.

[19] WHO consolidated guidelines on tuberculosis. Module 5: management of tuberculosis in children and adolescents. Geneva: World Health Organization; 2022. https://www.who.int/publications/i/item/9789240046764. (Last accessed 20 May 2023) 35404556

[20] National Department of Health. Guidelines for the management of tuberculosis in children. Pretoria, South Africa: Department of Health; 2013. https://knowledgehub.health.gov.za/system/files/elibdownloads/2023-04/National-Childhood-TB-Guidelines-2013-ZA.pdf. (Last accessed 21 July 2023)

[21] van Toorn R, Springer P, Laubscher JA, Schoeman JF (2012). Value of different staging systems for predicting neurological outcome in childhood tuberculous meningitis. Int J Tuberc Lung Dis.

[22] Figaji A, Fieggen G, Rohlwink U (2017) Hydrocephalus surgery in childhood tuberculous meningitis with hydrocephalus. In: Tuberculosis of the central nervous system: pathogenesis, imaging, and management. Springer International Publishing AG. 419–428

[23] Yadav YR, Yadav N, Parihar V, Ratre S, Bajaj J (2017) Role of Endoscopic Ventriculostomy in tuberculous meningitis with hydrocephalus. In: Tuberculosis of the central nervous system: pathogenesis, imaging, and management. Springer International Publishing AG. 429–446

[24] South Africa National Department of Health. Implementation of the universal teat and treat strategy for HIV positive patients and differentiated care for stable patients. Pretoria: South Africa National Department of Health. 2016. https://sahivsoc.org/Files/22%208%2016%20Circular%20UTT%20%20%20Decongestion%20CCMT%20Directorate.pdf. (Last accessed 29 April 2023)

[25] World Health Organization (WHO). Guideline on when to start antiretroviral therapy and on pre-exposure prophylaxis for HIV. 2015. https://apps.who.int/iris/bitstream/handle/10665/186275/9789241509565_eng.pdf. (Last accessed 25 May 2023) 26598776

[26] Misra UK, Kalita J, Bhoi SK, Singh RK (2016). A study of hyponatremia in tuberculous meningitis. J Neurol Sci.

[27] Karande S, Gupta V, Kulkarni M, Joshi A, Rele M (2005). Tuberculous meningitis and HIV. Indian J Pediatr.

[28] Topley JM, Bamber S, Coovadia HM, Corr PD (1998). Tuberculous meningitis and co-infection with HIV. Ann Trop Paediatr.

[29] Marais S, Pepper DJ, Marais BJ, Török ME (2010). HIV-associated tuberculous meningitis–diagnostic and therapeutic challenges. Tuberculosis (Edinb).

[30] Bang ND, Caws M, Truc TT, Duong TN, Dung NH, Ha DT (2016). Clinical presentations, diagnosis, mortality and prognostic markers of tuberculous meningitis in Vietnamese children: a prospective descriptive study. BMC Infect Dis.

[31] Grobbelaar M, van Toorn R, Solomons R (2018). Lumbar cerebrospinal fluid evolution in childhood tuberculous meningitis. J Child Neurol.

[32] Rohlwink UK, Donald K, Gavine B, Padayachy L, Wilmshurst JM, Fieggen GA (2016). Clinical characteristics and neurodevelopmental outcomes of children with tuberculous meningitis and hydrocephalus. Dev Med Child Neurol.

[33] Seipone ID, Singh R, Patel VB, Singh A, Gordon ML, Muema DM (2018). Tuberculous meningitis is associated with higher cerebrospinal HIV-1 viral loads compared to other HIV-1-associated meningitides. PLoS ONE.

[34] Rohlwink UK, Kilborn T, Wieselthaler N, Banderker E, Zwane E, Figaji AA (2016). Imaging features of the brain, cerebral vessels and spine in pediatric tuberculous meningitis with associated hydrocephalus. Pediatr Infect Dis J.

[35] Rohlwink UK, Mauff K, Wilkinson KA, Enslin N, Wegoye E, Wilkinson RJ (2017). Biomarkers of cerebral injury and inflammation in pediatric tuberculous meningitis. Clin Infect Dis.

[36] Clemente Morgado T, Kinsky M, Carrara H, Rothemeyer S, Semple P (2013). Prognostic value of computed tomography-evident cerebral infarcts in adult patients with tuberculous meningitis and hydrocephalus treated with an external ventricular drain. World Neurosurg.

[37] Chatterjee S (2011) Brain tuberculomas, tubercular meningitis, and post-tubercular hydrocephalus in children. J Pediatr Neurosci 6:S96–S100. 10.4103/1817-1745.85725 10.4103/1817-1745.85725 PMC3208909 22069437

[38] Bovijn L, Solomons R, Marais S (2019) Neurological TB in HIV. In: HIV and tuberculosis, a formidable alliance. Springer International Publishing AG. 295–334

[39] Lanzafame M, Vento S (2016). Tuberculosis-immune reconstitution inflammatory syndrome. J Clin Tuberc Other Mycobact Dis.

[40] van Toorn R, Rabie H, Dramowski A, Schoeman JF (2012). Neurological manifestations of TB-IRIS: a report of 4 children. Eur J Paediatr Neurol.

[41] Hall WA, Turgut AT, Turgut M (2017) Surgical therapy of tuberculosis of the nervous system and its covering. In: Tuberculosis of the central nervous system: pathogenesis, imaging, and management. Springer International Publishing AG. 401–417

[42] Goga A, Chirinda W, Ngandu NK, Ngoma K, Bhardwaj S, Feucht U (2018). Closing the gaps to eliminate mother-to-child transmission of HIV (MTCT) in South Africa: understanding MTCT case rates, factors that hinder the monitoring and attainment of targets, and potential game changers. S Afr Med J.

[43] Gulen ST, Turgut M, Gulec GU, Turgut AT, Akhaddar A (2017) Medical therapy. In: Tuberculosis of the central nervous system: pathogenesis, imaging, and management. Springer International Publising AG. 391–398

[44] Agrawal D, Gupta A, Mehta VS (2005). Role of shunt surgery in pediatric tubercular meningitis with hydrocephalus. Indian Pediatr.

[45] Lamprecht D, Schoeman J, Donald P, Hartzenberg H (2001). Ventriculoperitoneal shunting in childhood tuberculous meningitis. Br J Neurosurg.

[46] Kankane VK, Gupta TK, Jaiswal G (2016). Outcome of ventriculoperitoneal shunt surgery, without prior placement of external ventricular drain in Grades III and IV patients of tubercular meningitis with hydrocephalus: a single institution’s experience in the pediatric population and review of literature. J Pediatr Neurosci.

[47] Aranha A, Choudhary A, Bhaskar S, Gupta LN (2018). A randomized study comparing endoscopic third ventriculostomy versus ventriculoperitoneal shunt in the management of hydrocephalus due to tuberculous meningitis. Asian J Neurosurg.

[48] Figaji AA, Fieggen AG, Peter JC (2007). Endoscopy for tuberculous hydrocephalus. Childs Nerv Syst.

[49] Legaspi GD, Espiritu AI, Omar AT (2021). Success and complication rates of endoscopic third ventriculostomy for tuberculous meningitis: a systematic review and meta-analysis. Neurosurg Rev.

[50] Goyal P, Srivastava C, Ojha BK, Singh SK, Chandra A, Garg RK, Srivastava S (2014). A randomized study of ventriculoperitoneal shunt versus endoscopic third ventriculostomy for the management of tubercular meningitis with hydrocephalus. Childs Nerv Syst.

